# Apolipoprotein A-I mimetic peptide 4F suppresses tumor-associated macrophages and pancreatic cancer progression

**DOI:** 10.18632/oncotarget.21157

**Published:** 2017-09-22

**Authors:** Meiyu Peng, Qi Zhang, Yingnan Cheng, Shuyu Fu, Huipeng Yang, Xiangdong Guo, Jieyou Zhang, Lina Wang, Lijuan Zhang, Zhenyi Xue, Yan Li, Yurong Da, Zhi Yao, Liang Qiao, Rongxin Zhang

**Affiliations:** ^1^ Department of Immunology, School of Clinical Medicine, Weifang Medical University, Weifang, China; ^2^ Laboratory of Immunology and Inflammation, Department of Immunology, Key Laboratory of Immune Microenvironment and Diseases of Educational Ministry of China, Tianjin Key Laboratory of Molecular and Cellular Immunology, Tianjin Medical University, Tianjin, China; ^3^ Institute of Integrative Medicines for Acute Abdominal Diseases, Nankai Hospital, Tianjin, China; ^4^ Institute of Human Virology, Sun Yat-Sen University, Guangzhou, China; ^5^ Storr Liver Unit, Westmead Institute for Medical Research, the University of Sydney and Westmead Hospital, Westmead, New South Wales, Australia; ^6^ Laboratory of Immunology and Inflammation, School of Life Science and Biopharmaceutics, Guangdong Pharmaceutical University, Guangzhou, China

**Keywords:** pancreatic cancer, L-4F, inflammation, tumor-associated macrophages, STAT3

## Abstract

Pancreatic cancer is an aggressive malignancy that is unresponsive to conventional radiation and chemotherapy. Therefore, development of novel immune therapeutic strategies is urgently needed. L-4F, an Apolipoprotein A-I (ApoA-I) mimetic peptide, is engineered to mimic the anti-inflammatory and anti-oxidative functionalities of ApoA-I. In this work, H7 cells were orthotopically implanted in C57BL/6 mice and treated with L-4F. Then, pancreatic cancer progression and the inflammatory microenvironment were investigated *in vivo*. The cytotoxicity of L-4F toward H7 cells was assessed *in vitro*. Furthermore, we investigated the effects of L-4F on macrophage polarization by analyzing the polarization and genes of mouse bone marrow-derived macrophages *in vitro*. The results show that L-4F substantially reduced the tumorigenicity of H7 cells. L-4F inhibited inflammation by reducing the accumulation of inflammatory cells, such as IL-17A-, IL-4-, GM-CSF-, IL-1β-, and IL-6-producing cells and Th1 and Th17. Notably, L-4F also decreased the percentage of macrophages in tumor tissues, especially M2 macrophages (CD11b^+^F4/80^+^CD206^+^), which was also confirmed *in vitro*. Additionally, the expression of the M2 marker genes *Arg1*, *MRC1*, and *CCL22* and the inflammatory genes *IL-6*, *iNOS*, and *IL-12* was decreased by L-4F, indicating that L-4F prevents M2 type macrophage polarization. However, L-4F could not directly attenuate H7 cell invasion or proliferation and did not induce apoptosis. In addition, L-4F potently down-regulated STAT3, JNK and ERK signaling pathways but not affects the phosphorylation of p38 in RAW 264.7 cells. These results suggest that L-4F exhibits an effective therapeutic effect on pancreatic cancer progression by inhibiting tumor-associated macrophages and inflammation.

## INTRODUCTION

Pancreatic cancer is the fourth leading cause of cancer-related death in developed countries, with an overall 5-year survival rate below 5%. It is generally asymptomatic until it has reached an advanced stage and is typically unresponsive to conventional radiation and chemotherapy, resulting in a mortality rate of nearly 100% within 6 months of diagnosis [1]. Therefore, novel therapeutic strategies are urgently needed.

Apolipoprotein A-I (ApoA-I), the major protein component of high-density lipoprotein (HDL), reverses cholesterol transport by extracting and transferring cholesterol and phospholipids from peripheral cells to the liver for excretion. In addition to its antiatherogenic properties, ApoA-I also exhibits anti-inflammatory and anti-oxidant properties [2]. Reconstituted HDL has been shown to reduce inflammation by inhibiting cellular expression of vascular cell adhesion molecule-1 and intercellular adhesion molecule-1 in stimulated human coronary endothelial cells and reducing CD11b expression in circulating monocytes [3]. L-4F, an ApoA-I mimetic peptide, was engineered to mimic the anti-inflammatory and anti-oxidative functionalities of ApoA-I, which have shown positive effects for cancer treatment and for decreasing inflammation [4, 5]. It has been reported that L-4F suppresses tumor progression by inhibiting the expression and activity of hypoxia-inducible factor-1α and tumor angiogenesis in human ovarian cancer [6], reduces cell viability and proliferation in ovarian cancer cells, and decreases plasma levels of lysophosphatidic acid in colon cancer [7, 8]. It has also recently been found that L-4F inhibits LPS-mediated elevation of TNF-α and IL-6 in LPS-stimulated neutrophils [9].

The therapeutic effects of L-4F on pancreatic cancer and tumor immune microenvironments remain poorly understood. In this study, we aimed to explore whether L-4F can inhibit pancreatic cancer progression and to determine its cellular and molecular mechanisms.

## RESULTS

### L-4F attenuates the progression of pancreatic tumors in mice

**Figure 1:**
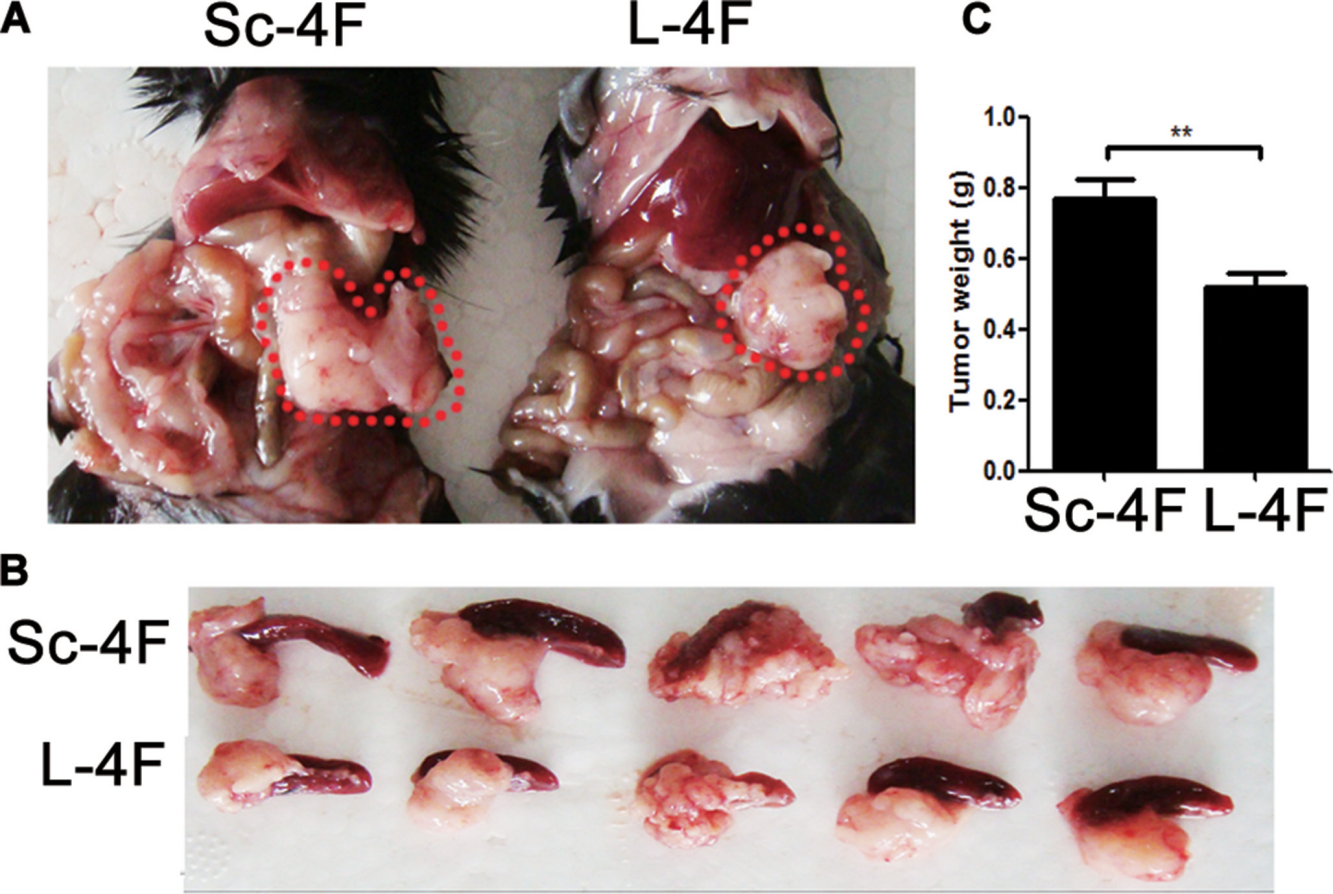
L-4F delays H7 tumor progression in mice H7 cells were injected into the pancreas. Mice were euthanized after 1 wk of L-4F or Sc-4F treatment. (**A**, **B**) Representative tumors from Sc-4F- or L-4F-treated mice; pancreatic cancer is outlined using a red dashed line. (**C**) Final tumor weights (^*^P < 0.05).

We first evaluated whether L-4F might exhibit suppressive effects on pancreatic cancer development in a mouse model. To accomplish this, C57BL/6 mice were implanted with H7 pancreatic cancer cells through direct injection into the pancreas. L-4F (10 mg/kg) or Sc-4F (10 mg/kg), a control peptide, was administered. As shown in Figure 1A–1C, compared with tumors in Sc-4F-treated mice, L-4F treatment significantly reduced both tumor size (Figure 1A, 1B) and tumor weight (0.77 g *vs* 0.52 g, P<0.01) (Figure 1C).

### L-4F did not inhibit migration, reduce proliferation or induce apoptosis of H7 cells

**Figure 2:**
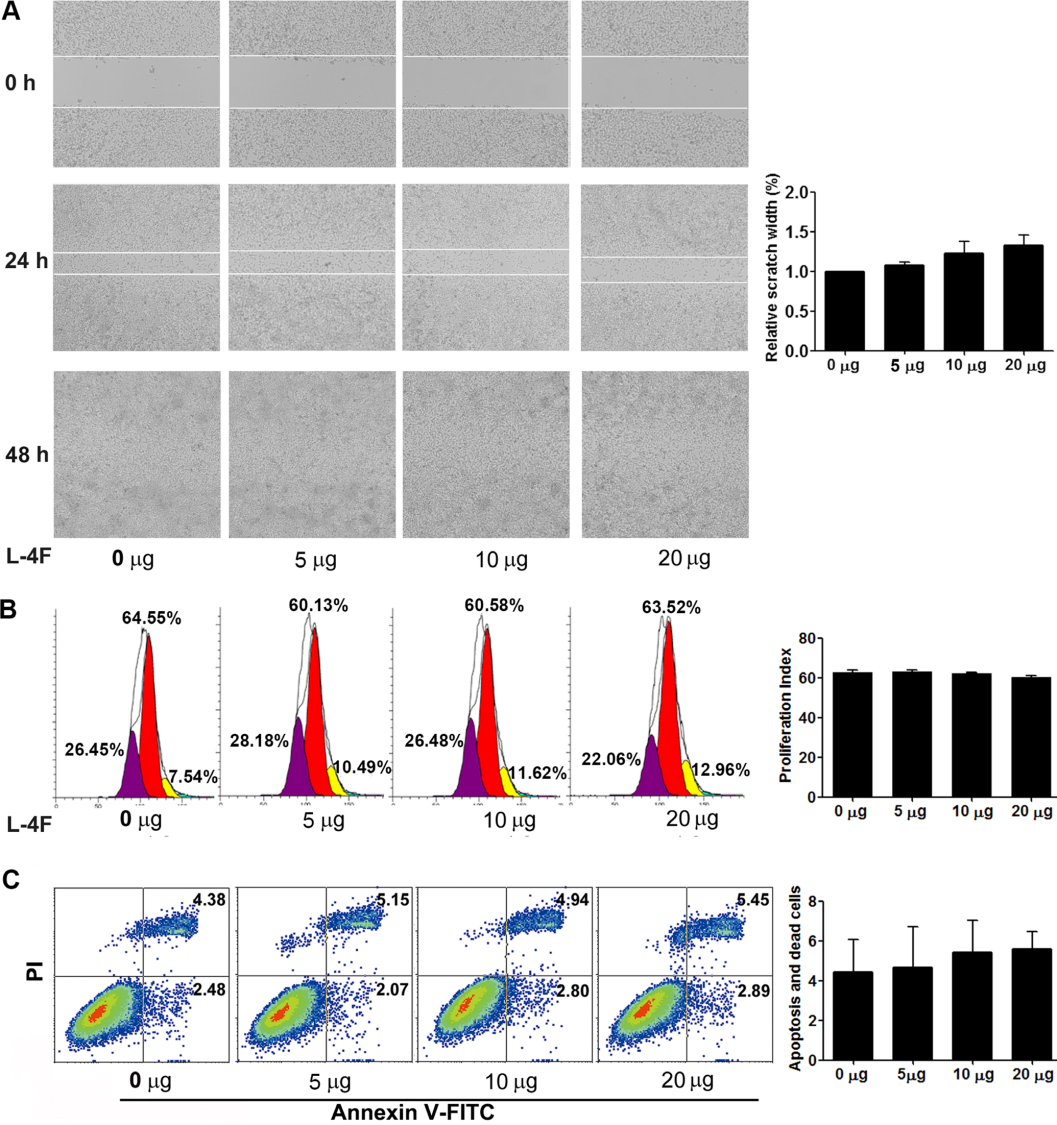
L-4F could not directly attenuate H7 cell invasion or proliferation and did not induce apoptosis H7 cells were treated with L-4F (0, 5, 10, or 20 μg/mL). (**A**) Representative images of wound healing in a scratch assay of H7 cells treated with L-4F at 0, 24 and 48 h after wounding. The distances between wound edges in three randomly chosen regions were normalized to 100% in untreated cells at 24 h. (**B**) One representative result from each experiment is shown: each peak represents the population of cells with reduced CFSE content due to cell division and the proliferative index (PI) at 48 h. (**C**) The percentage of apoptotic cells treated with L-4F at 48 h. Cells in the lower right quadrant were scored as “early apoptotic” (Annexin^+^/PI^-^), and cells in the upper right quadrant were scored as “necrotic/late apoptotic” (Annexin^+^/PI^+^).

H7 cells were treated with vehicle or the indicated concentration of L-4F (5, 10, or 20 μg/mL) and submitted to a wound healing assay. Wound width was photographed using light microscopy at 0, 24 and 48 h after scraping. As shown in Figure 2A, there were no clear differences in wound healing for the duration of L-4F treatment. As shown in Figure 2B, the proliferative index (PI) of the L-4F-treated cells was not obviously reduced compared to the untreated or low dose-treated cells (PI = 62.74, 63.17, 62.28, and 60.22 for 0, 5, 10, and 20 μg/mL, respectively, NS). In addition, compared with the untreated cells, the populations of early apoptotic, necrotic, and late apoptotic cells were not obviously changed in the L-4F-treated cells (4.43%, 4.68%, 5.44% and 5.88%, for 0, 5, 10, and 20 μg/mL, respectively, NS) (Figure 2C).

### L-4F decreases inflammatory cell infiltration in mice with pancreatic cancer

**Figure 3:**
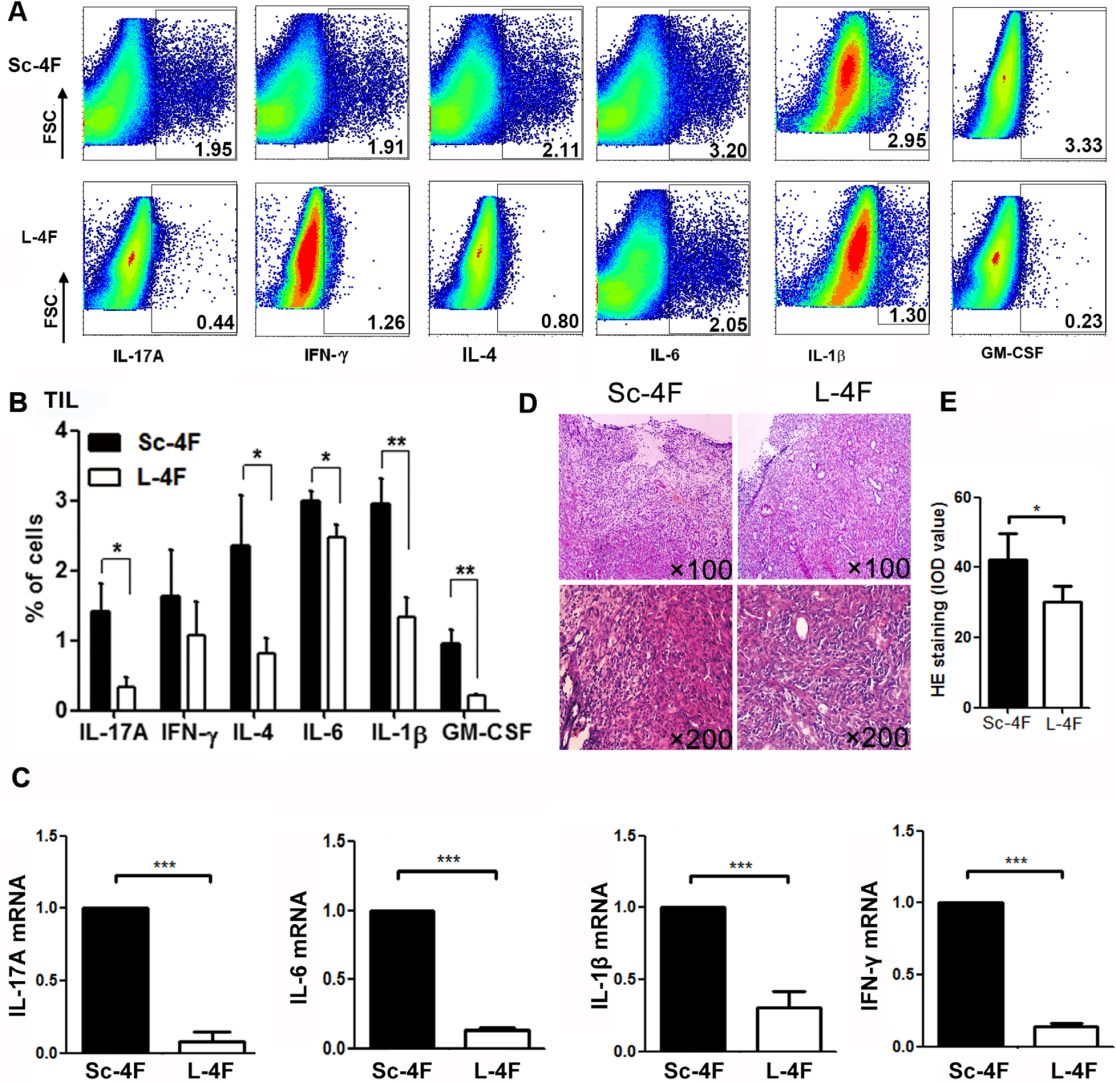
L-4F reduces inflammation in a mouse model of pancreatic cancer Tumors were collected from Sc-4F- or L-4F-treated mice. Single-cell suspensions were acquired, and the cytokines were immunostained as described in the Materials and Methods section. (**A**) One representative result from each experiment is shown. (**B**) The percentages of IL-17A-, IFN-γ-, IL-4-, IL-6-, IL-1β- and GM-CSF-producing cells among tumor infiltrating cells in tumor tissues (^*^P < 0.05, ^**^P < 0.01). (**C**) The mRNA levels of IL-17A, IFN-γ, IL-6, and IL-1β in tumor tissues. (**D**) H&E staining showing infiltration of inflammatory cells in tumor tissues. (**E**) Image-Pro Plus was used to quantify the relative IOD value of HE staining of inflammatory cells in tumor tissues (^*^P < 0.05).

**Figure 4:**
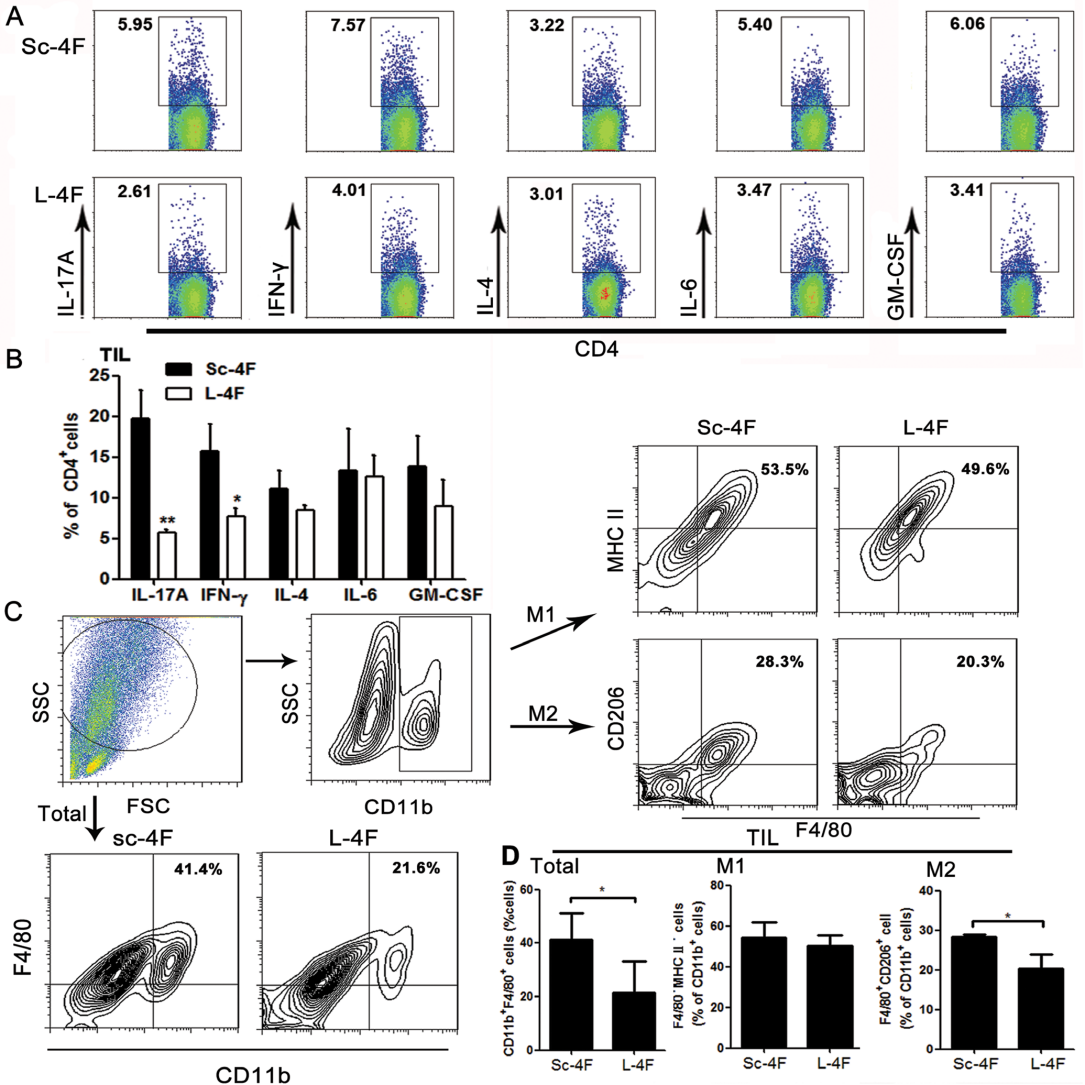
L-4F decreases Th17 cell, Th1 cell and TAM populations in a mouse pancreatic cancer model Tumors were collected from Sc-4F- or L-4F-treated mice. Single-cell suspensions were acquired and stained as described in the Materials and Methods section. (**A**) One representative result from each experiment is shown. (**B**) The percentages of IL-17A-, IFN-γ-, IL-4-, IL-6- and GM-CSF-producing cells among tumor infiltrating Th cells (^*^P < 0.05, ^**^P < 0.01). Th17 and Th1 cells represent two particular subsets of T helper lymphocytes characterized by production of IL-17 and IFN-γ cytokines, respectively. (**C**) The percentage of CD11b^+^F4/80^+^ cells among tumor infiltrating cells. The number of F4/80^+^ macrophages among the total CD11b^+^cells was quantified. (**D**) The blot shown was gated on CD11b^+^ cells, and an F4/80^+^MHC II^+^ and F4/80^+^CD206^+^ flow plot was used to identify M1 and M2 macrophages, respectively. The number of F4/80^+^MHC II^+^ and F4/80^+^CD206^+^ macrophages among the total CD11b^+^ cells was quantified (^*^P < 0.05).

As shown in Figure 3D, L-4F treatment obviously reduced inflammatory cell infiltration in tumor tissues collected from mice. Therefore, we further analyzed the percentages of IL-17A-, IL-6-, IFN-γ-, IL-4-, granulocyte macrophage colony stimulating factor (GM-CSF)- and IL-1β-producing cells in tumor-infiltrating cell populations from mice administered L-4F or Sc-4F.

Compared with the Sc-4F-treated group, in the mice treated with L-4F, the percentages of tumor-infiltrating cells producing IL-17A (1.41% *vs* 0.33%, *P <* 0.05), IL-4 (2.35% *vs* 0.81%, *P <* 0.05), IL-6 (2.99% *vs* 2.47%, *P <* 0.01), IL-1β (2.95% *vs* 1.34%, *P <* 0.01) and GM-CSF (0.96% *vs* 0.22%, *P <* 0.01) all significantly decreased, whereas the percentage of tumor-infiltrating cells producing IFN-γ (1.63%*vs* 1.07%, NS) did not exhibit significant changes (Figure 3A, 3B).

### L-4F decreases mRNA levels of inflammatory cytokines in mice with pancreatic cancer

To further confirm the anti-inflammatory effects of L-4F, we analyzed mRNA levels of the inflammatory cytokines IL-17A, IFN-γ, IL-6, and IL-1β in tumor tissues from Sc-4F- or L-4F-treated tumor-bearing mice. As shown in Figure 3C, L-4F substantially decreased the mRNA levels of the inflammatory cytokines *IL-17A*, *IFN-γ*, *IL-6* and *IL-1β* (average fold-change of 0.107 for *IL-17A*, 0.155 for *IFN-γ*, 0.069 for *IL-6*, 0.340 for *IL-1β*, *P<*0.001).

### L-4F decreases Th17 and Th1 cell populations in mice with pancreatic cancer

We further analyzed the percentages of IL-17A-, IL-6-, IFN-γ-, IL-4- and GM-CSF-producing Th cells (T helper cells) in tumor-infiltrating lymphocytes (TILs) from H7 tumor-bearing mice treated with Sc-4F or L-4F. Compared with the Sc-4F-treated mice, in the mice treated with L-4F, the percentages of tumor-infiltrating Th cells producing IL-17A (19.65% *vs* 5.65%, *P <* 0.05) and IFN-γ (15.72% *vs* 7.7%, *P <* 0.05) significantly decreased, whereas the percentages of tumor-infiltrating Th cells producing IL-4 (11.11% *vs* 8.51%, NS), IL-6 (13.29% *vs* 12.53%, NS), and GM-CSF (13.84% *vs* 9.0%, NS) did not exhibit significant changes (Figure 4A, 4B).

### L-4F inhibits the augmentation of tumor-associated macrophage populations in mice with pancreatic cancer

The activation status of macrophages in tumor tissue was also assessed using flow cytometry. Populations of M1 macrophages and M2 macrophages were determined using MHC II and CD206 (also called mannose receptor C type 1, Mrc1) as markers. Compared with the Sc-4F-treated group, the percentage of CD11b^+^F4/80^+^ macrophages (41.4% *vs* 21.6%, *P <* 0.05) significantly decreased (Figure 4C, 4D) in the L-4F treated group. Similarly, L-4F effectively inhibited the augmentation of F4/80^+^CD206^+^ M2 macrophage populations (28.3% *vs* 20.3%, *P<*0.01) (Figure 4C, 4D).

### L-4F suppresses M2 macrophage polarization and decreases the expression of macrophage-associated genes

**Figure 5:**
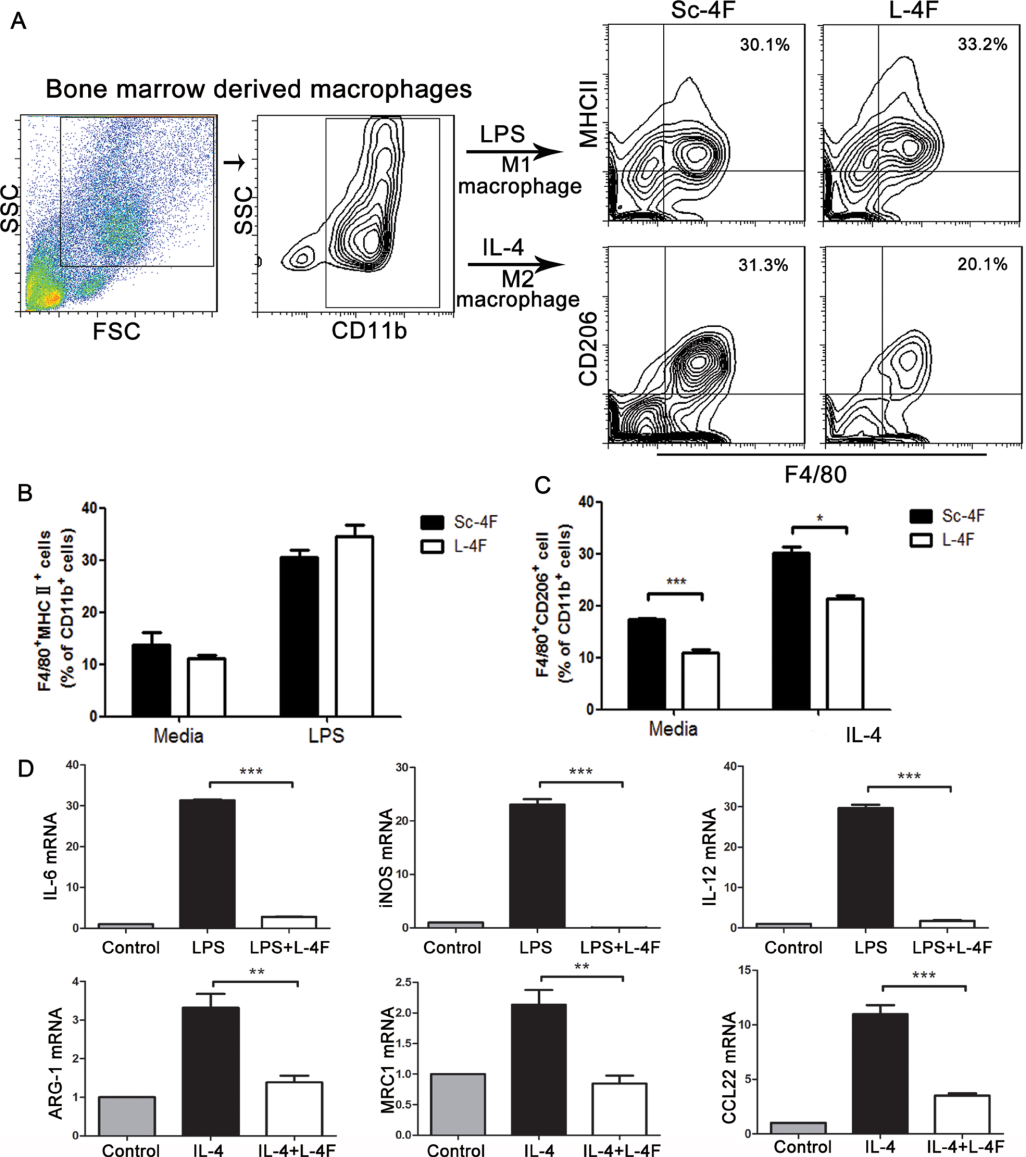
L-4F inhibits M2 macrophage polarization and decreases the expression of macrophage-associated genes BMDMs were exposed to LPS or IL-4 in the presence or absence of L-4F as described in the Materials and Methods section. (**A**) The blot shown was gated on CD11b^+^ cells, and an F4/80^+^MHC II^+^ and F4/80^+^CD206^+^ flow plot was used to identify M1 and M2 macrophages, respectively. (**B** and **C**) The number of F4/80^+^MHC II^+^ and F4/80^+^CD206^+^ macrophages among the total CD11b^+^ cells was quantified. (**D**) The expression of the M1 genes IL-6, iNOS and IL-12 and the M2 genes ARG-1, MRC1, and CCL22 was analyzed using qPCR (^*^P < 0.05, ^***^P < 0.001).

To further examine the effect of L-4F in macrophages *in vitro*, we differentiated mouse bone marrow-derived macrophages to M1 or M2 macrophages using LPS (for M1) or IL-4 (for M2) in the presence of Sc-4F or L-4F. M1-like macrophages are defined here as CD11b^+^ F4/80^+^MHC II^+^ cells, while M2-like macrophages are defined as CD11b^+^ F4/80^+^CD206^+^ cells. As shown in Figure 5A–5C, the number of M2 macrophages significantly decreased in the group treated with L-4F compared to the Sc-4F-treated group (31.3% *vs* 20.1%, *P<*0.05). In contrast, there were no significant changes in M1 macrophage populations. These results indicate that L-4F treatment inhibits the augmentation of M2 macrophage populations *in vitro*.

The M1 and M2 phenotypes were further confirmed by gene expression analysis. To accomplish this, we measured the expression of several M1 cytokines (*IL-6, iNOS,* and *IL-12*) and M2 molecules (*ARG-1, MRC1,* and *CCL22*). Compared to undifferentiated macrophages, M1- or M2-differentiated macrophages showed significant upregulation of M1- or M2-specific inflammatory genes, and expression of these genes was substantially decreased in the L-4F-treated group (average fold-change of 0.0042 for IL-6, 0.0014 for *iNOS*, 0.0842 for *IL-12*, 0.415 for *ARG-1,* 0.82 for *MRC1,* 0.317 for *CCL22,* , *P <* 0.001) (Figure 5D).

### L-4F down-regulated STAT3 and MAPK signaling pathways in lipopolysaccharide-treated RAW 264.7 cells

**Figure 6:**
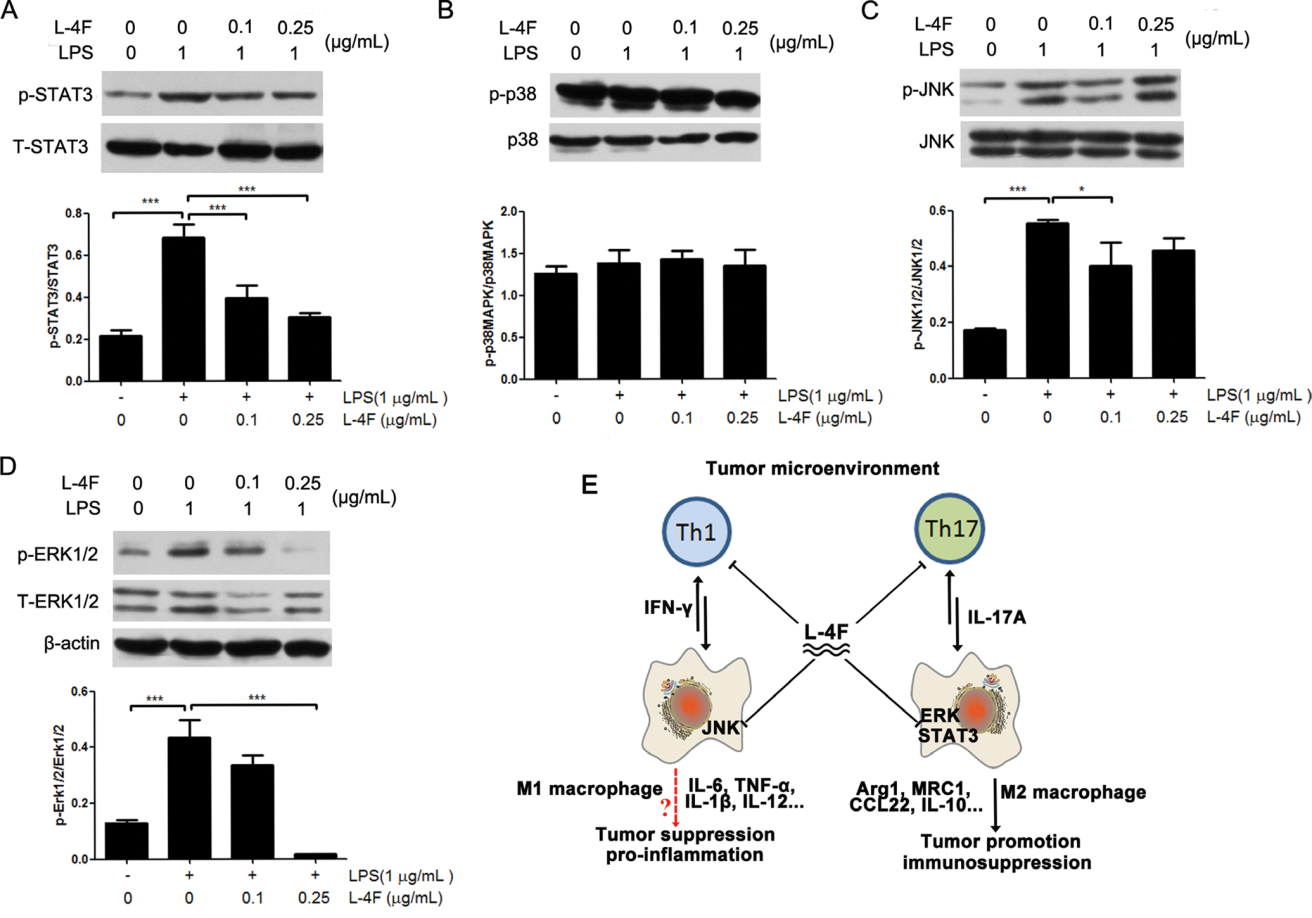
L-4F down-regulated STAT3 and MAPK signaling pathways in lipopolysaccharide-treated RAW 264.7 cells RAW 264.7 macrophages were treated with the indicated concentrations of L-4F and then stimulated with LPS. The levels of pSTAT3, pp38, pJNK and pERK were analyzed by western blotting, and the results are shown in (**A**–**D**) respectively (^*^P < 0.05, ^**^P < 0.01). (**E**) Regulation mechanism of L-4F in the tumor microenvironment. The tumor microenvironment (TME) is a highly dynamic system that adopts a variety of activation states in response to inflammation. L-4F is a negative regulator of Th1 and Th17 cells as well as macrophages in the TME. Specifically, L-4F is a crucial suppressor of M2-type macrophage polarization and inhibits IFN-γ and IL-17A production in response to the TME. Mechanistically, L-4F suppresses the STAT3, ERK and JNK signaling pathways in macrophages. These pathways are critical for tumor progression and eventually lead to fatal outcomes.

We further evaluated the effect of L-4F on activation of STAT3 and LPS-induced activation of the MAPK pathway in RAW 264.7 cells. As shown in Figure 5, compared to the LPS control group, L-4F treatment significantly suppressed the phosphorylation of STAT3 (Figure 6A) and inhibited the phosphorylation of JNK and ERK1/2 (Figure 6C and 6D) in LPS-treated RAW 264.7 cells in a low dose-dependent manner. However, L-4F had no obvious effect on the p38MAPK (Figure 6B) signaling pathway.

## DISCUSSION

It has been reported that L-4F can suppress tumor progression in ovarian cancer and colon cancer by reducing cell viability and proliferation [7, 8]. In addition, L-4F was shown to significantly reduce cell viability in the human ovarian cancer cell lines SKOV3, OV2008, and A2780 by binding and removing lysophosphatidic acid, which is a well-known activator of proliferation in cancer cells. Despite these promising results, however, L-4F was not effective in the OVCAR5 cell line [8]. These reports indicate that L-4F can suppress cancer growth, but not all tumor cells are sensitive to L-4F. In our *in vivo* study, we found that L-4F substantially reduced the tumorigenicity of H7 pancreatic tumors in a mouse model. However, our *in vitro* results demonstrated that L-4F could not suppress the invasion (Figure 2A) or proliferation (Figure 2B) capacity of H7 cells (or Panc1 cells, Supplementary Figure 1) and did not induce cell apoptosis in these cells (Figure 2C). Therefore, we conclude that L-4F has no significant direct anti-tumor effect in the mouse pancreatic cancer H7 cell line, leaving the biological basis underlying the ability of L-4F to help mice resist pancreatic cancer unresolved.

Inflammation, such as in chronic pancreatitis, has been shown to represent a major risk factor for pancreatic cancer [10]. Inflammation plays a critical role in tumorigenesis: it can increase the proliferation and viability of malignant cells, promote angiogenesis and metastasis, and subvert adaptive immune responses [11]. Cytokines derived from immune cells or cancer cells play important roles in tumor immunoediting. In particular, IL-6 has emerged as an important factor in the modulation of cancer-associated inflammation [12], and pancreatic cancer cells highly express this cytokine [13]. Pancreatic tumor cells also release the pro-inflammatory cytokines IL-1β and TNF-α, which play a critical role in the malignancy of pancreatic ductal adenocarcinoma (PDA) [14, 15]. Serum levels of GM-CSF in pancreatic cancer patients are also significantly higher than in healthy individuals [16], and increased expression of the inflammatory cytokines IFN-γ, CXCL1 and CXCL2 were observed in a mouse model of spontaneous pancreatic cancer [17].

Several studies have determined that L-4F possesses effective anti-inflammatory properties. For example, L-4F stimulates HDL anti-inflammatory activity and inhibits LDL pro-inflammatory activity in the plasma of patients with end-stage renal disease [4]. L-4F also significantly decreased serum interleukin (IL)-6, TNF-α and IL-1β in obese mice [18]. Moreover, L-4F was shown to inhibit LPS-induced inflammatory responses by reducing the synthesis of cytokines, chemokines, and adhesion molecules [5]; reduce pro-inflammatory gene expression in activated human coronary artery endothelial cells and macrophage foam cells; and prevent reactive oxygen species formation in activated neutrophils [19]. In this study, we investigated the anti-inflammatory effect of L-4F in a mouse pancreatic cancer model. Our results show that L-4F treatment significantly inhibited tumor progression (Figure 1A–1C), decreased inflammatory cell infiltration in tumor tissues (Figure 3D), and reduced percentages of IL-17A-, IL-6-, GM-CSF- and IL-1β-producing cells to varying degrees in tumor tissues (Figure 3A, 3B). Moreover, L-4F decreased mRNA levels of the inflammatory cytokines IL-17A, IFN-γ, IL-6, and IL-1β (Figure 3C). These results indicate that L-4F suppresses pancreatic cancer progression through inhibition of inflammation by decreasing the infiltration of pro-inflammatory and inflammatory cytokine-producing cells in mice with pancreatic cancer.

Th cells, including Th1, Th2, and Th17 cells, can exert both tumor-suppressive and tumor-promoting effects [20, 21]. There is evidence that the processes of tumor promotion, progression, and metastasis are governed by select Th cell subsets, including IFN-γ-producing Th1 cells [22], IL-4-producing Th2 cells [15, 23] and IL-17-producing Th17 cells [24]. In this study, we investigated changes in Th1, Th2 and Th17 cell subpopulations by detecting the cytokines IFN-γ, GM-CSF, IL-4, IL-6 and IL-17A in TILs. We found that only the percentages of IL-17A- and IFN-γ-producing Th cells decreased (Figure 4A, 4B). Therefore, we conclude that L-4F inhibited pancreatic cancer progression by decreasing the infiltration of Th17 and Th1 inflammatory cells, but not other Th cell subpopulations, in a mouse model of pancreatic cancer.

Th cells respond to inflammation to maximize cytotoxicity against cancer cells in association with macrophages [25]. In addition, macrophages play a key role in the immune response and are the main component of inflammatory cell populations in solid tumors. As such, they are key regulators of the link between inflammation and various types of cancer. Macrophage depletion has been shown to successfully limit tumor growth, metastasis and angiogenesis, thereby leading to a better response to conventional therapy in metastatic breast cancer and colitis-associated cancer [26, 27]. Macrophages are categorized into “classically activated” M1 and “alternatively activated” M2 subtypes. M1 macrophages express high levels of pro-inflammatory cytokines (TNF-α, IL-6, and IL-12) and are capable of killing pathogens and priming anti-tumor immune responses. By contrast, M2 or “alternatively” activated macrophages show increased expression of the anti-inflammatory cytokine IL-10 as well as scavenger receptor A and arginase. M2-polarized macrophages have been shown to be significantly more abundant in primary PDA samples [28], and the presence of M2-polarized macrophages in the stroma is strongly correlated with tumors located in the tail and body of the pancreas [29]. Most tumor-associated macrophages (TAMs) are considered to have an M2 phenotype and to promote tumor angiogenesis and tissue remodeling [30]. TAMs are also an important source of cytokines [29]. Functionally, TAMs promote tumor growth and may be obligatory for promoting angiogenesis, enhancing tumor cell migration and invasion, and suppressing anti-tumor immune responses [8]. TAMs are also immunosuppressive, preventing tumor cells from being attacked by natural killer and T cells during tumor progression and after recovery from chemo- or immunotherapy [31]. The balance between pro-inflammatory and anti-inflammatory cytokines in the tumor microenvironment finely regulates macrophage polarization and activity [32]. M1 macrophages are activated by IFN-γ and LPS, whereas M2 macrophages arise in response to IL-4, IL-10 and IL-17A [33, 34]. Furthermore, higher numbers of M2-polarized TAMs are associated with an increased risk of lymph node metastasis, neural invasion, and chemoresistance in PDA and hence a worse prognosis and reduced survival [35, 36].

Therefore, targeting TAMs provides an opportunity for prevention and treatment of pancreatic cancer [37]. In the present study, we elucidated the effect of L-4F on TAMs using a pancreatic mouse model. Our results show (Figure 4C, 4D) that L-4F treatment augmented CD11b^+^F4/80^+^ macrophage populations and significantly decreased CD11b^+^F4/80^+^CD206^+^ M2-macrophage populations. This finding was further demonstrated in the *in vitro*, where IL-4-induced M2 polarization was directly reversed by L-4F (Figure 5A). Furthermore, L-4F led to down-regulation of IL-17A and IL-4 (Figure 3), suggesting that, in addition to its direct anti-inflammatory effect, L-4F can change the phenotypes of TAMs in pancreatic tumors by altering the microenvironment.

Increasing evidence indicates that signal transducer and activator of transcription 3 (STAT3) and mitogen-activated protein kinase (MAPK) signaling pathways are closely associated with inflammatory processes and play major roles in inflammation and cancer [38, 39]. Moreover, the major pathways of macrophage polarization have been outlined and reviewed [40]. In brief, M1 macrophage polarization results from exposure to LPS and activation of NF-κB and STAT1. In contrast, STAT3 and MAPK activation results in M2 macrophage polarization, which is associated with immune suppression and tumor progression [41]. The MAPK family of proteins includes ERK, p38, and JNK [42]. Some studies have reported that ApoA-I can inhibit the LPS-induced activation of JNK in mouse macrophage RAW264.7 cells [43] and can inhibit the production of IFN-γ and IL-6 in mouse serum [44]. In addition, ApoA-I inhibited STAT3, which plays a major role in suppressing LPS-induced production of the inflammatory cytokine IL-6, in a mouse model of colitis-associated carcinogenesis [45]. Here, our results demonstrated that L-4F could not inhibited the IFN-γ producing cells in tumor tissues and L-4F inhibited phosphorylation of LPS-induced JNK in mouse RAW264.7 macrophages, but not inhibit p38MAPK. On the other hand, L-4F inhibited both the cytokines (IL-4 and IL-17A) producing cells in tumor tissues and the important signaling molecules (phosphorylation of ERK and STAT3). These results indicated that L-4F could inhibit all of the key regulators of M2 macrophage, but not M1 macrophage. STAT3 signaling in macrophage cells is the potential mechanism that L-4F regulation.

In conclusion, we have demonstrated that L-4F substantially inhibits pancreatic cancer progression mostly by exerting anti-inflammatory activity. Additionally, macrophage targeting is a key component of the anti-inflammatory and anti-tumor properties of L-4F. Notably, L-4F prevents M2 macrophage differentiation through inhibition of the STAT3 and MAPK pathways in macrophages. Thus, L-4F plays a critical role in regulating the tumor microenvironment (Figure 6E). The current results strengthen the rationale for future validation of ApoA-I-mimetic peptides (such as L-4F), which may serve as adjuvant therapeutic agents for the treatment of pancreatic cancer.

## MATERIALS AND METHODS

### Cell lines

The highly metastatic mouse pancreatic cancer H7 cell line was established using an *in vivo* selection method described by Wang et al. [46]. H7 and RAW 264.7 macrophages were cultured in Dulbecco’s modified Eagle’s medium (DMEM) containing 10% fetal bovine serum (FBS), 100 mg/mL penicillin and 100 mg/mL streptomycin (Gibco) in a humidified atmosphere of 5% CO_2_ at 37°C.

### Animals and tumor model

Six- to eight-week-old female C57BL/6 mice were purchased from the Academy of Military Medical Science of China and were acclimated for 7 days in the laboratory before experimentation. All of the experiments were performed in accordance with university guidelines for animal care and were approved by the Animal Ethics Committee of Tianjin Medical University.

Mouse abdomens were prepped with betadine solution, and an approximately 1-cm-wide incision was made in the left upper quadrant of each abdomen. The tip of the pancreatic tail was grasped gently, and the pancreas and spleen were externalized in a lateral direction to fully expose them. A needle was inserted into the tail of the pancreas and positioned in the pancreatic head region. A 27-gauge needle was then used to slowly inject 1×10^6^ H7 cells suspended in 50 μL of PBS. The spleen was then returned to the appropriate position in the abdomen, and the skin and peritoneum were closed with 3–0 vicryl sutures. Subgroups of mice received either vehicle (50 mM ammonium bicarbonate, pH = 7.0, containing 0.1 mg/mL Tween-20) containing L-4F (the peptide Ac-D-W-F-K-A-F-Y-D-K-V-A-E-K-F-K-E-A-F-NH_2_ was synthesized from all L-amino acids, 10 mg/kg, n = 8) or vehicle containing scrambled peptide (Sc-4F) (containing the same amino acids as the 4F peptide but arranged in the sequence Ac-D-W-F-A-K-D-Y-F-K-K-A-F-V-E-E-F-A-K-NH2, 10 mg/kg, *n* = 8) by daily intraperitoneal injection beginning on day 3. All of the mice were euthanized after one week of treatment, and the tumor tissues were collected for further study.

### Histological staining

Tumor tissues were embedded in paraffin after being fixed in 4% (weight/volume) paraformaldehyde and were cut into 8-μm-thick sections. Paraffin sections were stained with hematoxylin and eosin (H&E).

### Isolation of immunocytes from tumor tissues

The tumor tissues were minced into small pieces and digested with 0.05 mg/mL each of type-IV collagenase, hyaluronidase and DNase I (Sigma) for 30 min at 37°C. Single-cell suspensions were obtained by grinding the digested tissues and filtering them through a 40-μm cell strainer (BD Biosciences). Immunocytes were isolated using Ficoll density gradient centrifugation [47].

### Generation of macrophages and *in vitro* assays

Macrophages was generated as previously described [27]. Briefly, bone marrow cells were isolated from the femurs and tibias of C57BL/6 mice and then seeded in cell culture dishes and cultured with complete DMEM supplemented with 10% FBS, 1% penicillin-streptomycin, and 20 ng/mL recombinant mouse M-CSF (PeproTech) at 37°C in a CO_2_ incubator for 5 days to differentiate into macrophages. The cells were washed twice with phosphate-buffered saline (PBS) every other day, and fresh medium was added. On day 6, nonadherent cells were removed and adherent cells were cultured with 20 ng/mL mouse recombinant IL-4 (PeproTech) or 1 μg/mL LPS (Sigma) for 24 h, either with or without L-4F (0.25 μg/mL). The differentiated, adherent, live macrophage population was detached from the plate with a solution containing trypsin (Gibco), and the cells were processed for phenotypic characterization. Based on the specific expression of a number of surface markers, including CD11b, F4/80, MHC II and CD206, the cells were sorted using flow cytometry.

### Flow cytometric analysis

The freshly isolated immunocytes were stained with antibodies for 30 min at 4°C, washed and analyzed on a BD FACSCalibur flow cytometer (BD Biosciences). The following monoclonal anti-mouse antibodies were used: anti-CD11b-FITC (or anti-CD11b-PE), anti-F4/80-APC, anti-MHC II-PE and anti-CD206-FITC (eBioscience).

For intracellular cytokine staining, the freshly isolated immunocytes were resuspended in complete RPMI 1640 medium (Gibco) supplemented with 2 mM L-glutamine, 100 IU penicillin, 100 mg/mL streptomycin, and 4.5 × 10^–5^ M 2-mercaptoethanol. The cells were stimulated with 50 ng/mL phorbol 12-myristate 13-acetate (PMA), 1 μg/mL ionomycin (Enzo Life Sciences, Farmingdale, USA) and 3 μg/mL brefeldin A (eBioscience) for 5 h. Then, the cells were fixed and permeabilized with Cytofix/Cytoperm buffers for 20 min at 4°C and washed with permeabilization wash buffer (BD Biosciences). Fc receptors were blocked with 2% rat serum and 10% bovine serum albumin prior to intracellular cytokine staining. Next, the cells were stained with rat anti-mouse IFN-γ-PE, IL-17A-PE, IL-6-FITC, IL-4-FITC, GM-CSF-PE and IL-1β-Percp (eBioscience). Finally, the cells were analyzed using a FACSCalibur flow cytometer (BD Biosciences).

Controls for nonspecific staining were monitored using isotype-matched mAbs, and nonspecific staining was always subtracted from the specific staining results. The acquired data were analyzed using CellQuest and WinMDI 2.9 software.

### Quantitative real-time PCR

Total RNA was extracted using TRIzol reagent according to the manufacturer’s instructions (Invitrogen). The RNA was converted into cDNA with random hexamers and M-MLV reverse transcriptase (Invitrogen). *GAPDH* was used as an internal control. The sequences of the primers used for qPCR are as follows: *IL-17A,* (F) 5′-CTACCTCAACCGTTCCAC-3′ and (R) 5′-CACCCACCAGCATCTTCT-3′); *IFN-γ,* (F) 5′-ATGAACGCTACACACTGCATC-3′ and (R) 5′-CCATCCTTTTGCCAGTTCCTC-3′); *IL-6,* (F) 5′-TAGTCCTTCCTACCCCAATTTCC-3′ and (R) 5′-TTGGTCCTTAGCCACTCCTTC-3′); *IL-1β,* (F) 5′-GCAACTGTTCCTGAACTCAACT-3′ and (R) 5′-ATCTTTTGGGGTCCGTCAACT-3′); *Argl,* (F) 5′-CTCCAAGCCAAAGTCCTTAGAG-3′ and (R) 5′-AGGAGCTGTCATTAGGGACATC-3′); *MRC1,* (F) 5′-CTCTGTTCAGCTATTGGACGC-3′ and (R) 5′-CGGAATTTCTGGGATTCAGCTTC-3′); *CCL22,* (F) 5′-CTCTGCCATCACGTTTAGTGAA-3′ and (R) 5′-GACGGTTATCAAAACAACGCC-3′); *iNOS,* (F) 5′-GTTCTCAGCCCAACAATACAAGA-3′ and (R) 5′-GTGGACGGGTCGATGTCAC-3′); *IL-12,* (F) 5′-ACTCTGCGCCAGAAACCTC-3′ and (R) 5′-CACCCTGTTGATGGTCACGAC-3′); and *GAPDH,* (F) 5′-AGGTCGGTGTGAACGGATTTG-3′ and (R) 5′-TGTAGACCATGTAGTTGAGGTCA-3′). Quantitative real-time PCR was performed with SYBR Green mix (Newbio), and the data are displayed as 2^-ΔCt^ values and are representative of at least three independent experiments.

### Western blotting

RAW 264.7 macrophages were plated in 6-well plates (4 × 10^5^/well) and cultured in 2 mL of DMEM for 4 h. The cultures were washed to remove non-adherent cells and then incubated with 2 mL of complete DMEM for 20 h. The culture medium was replaced with DMEM for 30 min to allow the cells to adjust. (i) To induce an inflammation model, 1 μg/mL LPS (Sigma) was added. After 24 h of stimulation with LPS, the cells were treated with the indicated concentrations of L-4F (0, 0.1, or 0.25 μg/mL) for 12 h, and the levels of pp38, pJNK and pERK were analyzed. (ii) Cells were stimulated by LPS and treated with the indicated concentrations of L-4F (0, 0.1, or 0.25 μg/mL) for 1 h, and the levels of pSTAT3 were analyzed.

Western blot procedure: Briefly, cells were lysed in radio-immunoprecipitation assay buffer containing the phosphatase and protease inhibitors phenylmethanesulfonyl fluoride and aprotinin (Sigma), and protein was collected. Then, the protein was separated by SDS-PAGE, transferred to PVDF membranes (Roche) and probed with the indicated primary antibodies (phosphorylated STAT3, phosphorylated p38, phosphorylated JNK and phosphorylated ERK; Cell Signaling Technology). The antibody-antigen complexes were detected using a Chemiluminescent HRP substrate kit (Millipore) according to the manufacturer’s protocols.

### Wound healing assay

H7 cells were seeded into 6-well plates and cultured in complete DMEM. Upon reaching approximately 70% confluence, the medium was replaced with serum-free medium. Following an overnight incubation, the cells reached at least 95% confluence, forming a confluent monolayer. A linear scratch was made using a 10-μL micropipette tip. The cells were further cultured in DMEM containing 2% FBS and the indicated concentrations of L-4F (0, 5, 10, or 20 μg/mL). Wound width was photographed using light microscopy (40×) at 0, 24 and 48 h. For evaluation of wound closure, three randomly selected points along each wound were marked. Each experiment was performed in triplicate. The measurements were obtained by measuring the distance between the wound edges using Image J software.

### Cell proliferation assay

Cells were starved overnight, and cell proliferation (cell division) was assayed using carboxyfluorescein succinimidyl ester (CFSE) labeling (Invitrogen). Briefly, the cells were trypsinized, washed twice in sterile PBS and resuspended in 2 mL of DMEM. CFSE was applied at a final concentration of 5 μM, and the cells were incubated at 37°C for 10 min in the dark. To quench the labeling, the cells were washed twice with complete DMEM. The labeled cells were treated with vehicle or the indicated concentrations of L-4F (5, 10, or 20 μg/mL) for 48 h in complete DMEM. Next, the cells were trypsinized and washed in PBS. Finally, the cells were assessed for labeling using a FACSCalibur flow cytometer. The acquired data were analyzed using CellQuest and Modfit software (BD Biosciences).

### Cellular apoptosis assay

Cells were treated with vehicle or the indicated concentrations of L-4F (5, 10, or 20 μg/mL) in complete medium for 48 h. After the indicated treatments, apoptosis was assessed using an Annexin V/PI assay kit (Invitrogen) according to the manufacturer’s protocol. The acquired data were analyzed using CellQuest software.

### Statistical analysis

All of the results were derived from at least three independent experiments. The data represent the mean values ± the standard deviation (SD). Comparisons between two groups were performed using Student’s unpaired *t*-test or one-way analysis of variance for comparison of three or more groups as indicated. Statistical analysis was performed using SPSS 10.0 software. *P* < 0.05 was considered statistically significant, and *P* ≥ 0.05 was not statistically significant (NS).

## SUPPLEMENTARY MATERIALS FIGURE



## Abbreviations

ApoA-I: Apolipoprotein A-I
HDL: high-density lipoprotein
DMEM: Dulbecco’s modified Eagle’s medium
FBS: fetal bovine serum
H&E: hematoxylin and eosin
PBS: phosphate-buffered saline
PMA: phorbol 12-myristate 13-acetate
GM-CSF: granulocyte macrophage colony stimulating factor
Th cells: T helper cells
TILs: tumor-infiltrating lymphocytes
Mrc1: mannose receptor C type 1
PDA: pancreatic ductal adenocarcinoma
TAMs: tumor-associated macrophages
STAT3: signal transducer and activator of transcription 3
MAPK: mitogen-activated protein kinase
