# Supplementary material for: Apolipoprotein A-I mimetic peptide 4F suppresses tumor-associated macrophages and pancreatic cancer progression
Source: Oncotarget. 2017 Sep 22;8(59):99693–706. doi: 10.18632/oncotarget.21157 (PMC5725125; doi:10.18632/oncotarget.21157)
Supplement: Supplementary file 1 [file oncotarget-08-99693-s001.pdf]

Apolipoprotein A-I mimetic peptide 4F suppresses tumor-associated macrophages and pancreatic cancer progression

SUPPLEMENTARY MATERIALS

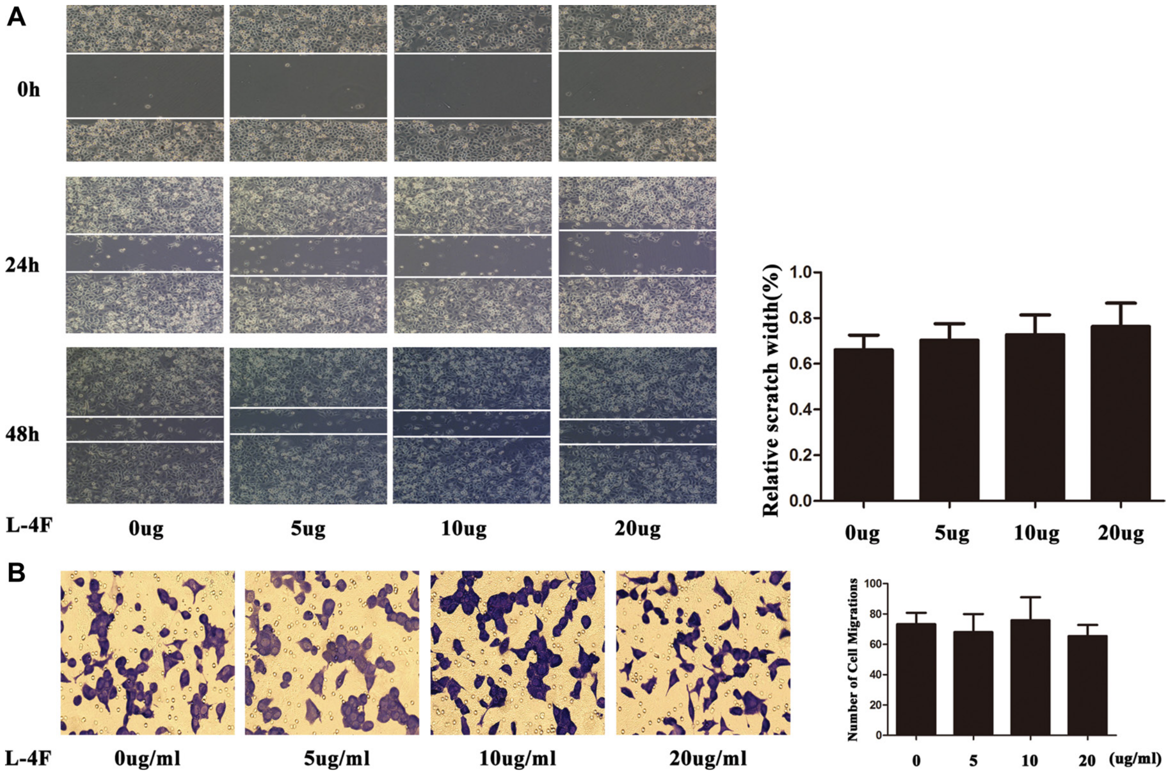

Supplementary Figure 1: L-4F could not directly attenuate Panc1 cell invasion or proliferation. Panc1 cells were treated with L-4F (0, 5, 10, or 20 µg/mL). (**A**) Representative images of wound healing in a scratch assay of Panc1 cells treated with L-4F at 0, 24 and 48 h after wounding. The distances between wound edges in three randomly chosen regions were normalized to 100% in untreated cells at 24 h. (**B**) Representative images of transwell membranes stained with crystal violet shows similar number of cells after L-4F treatment (0, 5, 10, or 20 µg/mL).
